# Changes in Cannabinoid Receptors, Aquaporin 4 and Vimentin Expression after Traumatic Brain Injury in Adolescent Male Mice. Association with Edema and Neurological Deficit

**DOI:** 10.1371/journal.pone.0128782

**Published:** 2015-06-03

**Authors:** Ana Belen Lopez-Rodriguez, Estefania Acaz-Fonseca, Maria-Paz Viveros, Luis M. Garcia-Segura

**Affiliations:** 1 Department of Animal Physiology (II), Biology Faculty, Complutense University of Madrid, Madrid, Spain; 2 Instituto Cajal, Consejo Superior de Investigaciones Cientificas (CSIC), Madrid, Spain; Martin Luther University, GERMANY

## Abstract

Traumatic brain injury (TBI) incidence rises during adolescence because during this critical neurodevelopmental period some risky behaviors increase. The purpose of this study was to assess the contribution of cannabinoid receptors (CB1 and CB2), blood brain barrier proteins (AQP4) and astrogliosis markers (vimentin) to neurological deficit and brain edema formation in a TBI weight drop model in adolescent male mice. These molecules were selected since they are known to change shortly after lesion. Here we extended their study in three different timepoints after TBI, including short (24h), early mid-term (72h) and late mid-term (two weeks). Our results showed that TBI induced an increase in brain edema up to 72 h after lesion that was directly associated with neurological deficit. Neurological deficit appeared 24 h after TBI and was completely recovered two weeks after trauma. CB1 receptor expression decreased after TBI and was negatively correlated with edema formation and behavioral impairments. CB2 receptor increased after injury and was associated with high neurological deficit whereas no correlation with edema was found. AQP4 increased after TBI and was positively correlated with edema and neurological impairments as occurred with vimentin expression in the same manner. The results suggest that CB1 and CB2 differ in the mechanisms to resolve TBI and also that some of their neuroprotective effects related to the control of reactive astrogliosis may be due to the regulation of AQP4 expression on the end-feet of astrocytes.

## Introduction

Traumatic brain injury (TBI) is the result of a mechanical insult to the brain that produces hematoma, hemorrhage, contusion and disruption of the blood brain barrier (BBB), which leads to brain edema formation [1]. The incidence of TBI varies with age, presenting an increase during adolescence [2]. One of the reasons why TBI rises in adolescence is because during this period, growth, freedom feeling and risky behaviors increase [3]. Adolescent rodents also show elevated levels of novelty seeking [4], impulsivity and risk-taking behavior [5]. The causes for TBI also vary with age. Among adolescents, the leading cause is motor accidents and falls [2]. This kind of accidents mostly induce close-head injuries that represent a high percentage of TBI patients (85–89%) [6,7] and their lesions present a high variability, complexity and unpredictable prognosis. Closed-head trauma animal models have been developed to understand the physiopathology of TBI, which in the case of developing brains such as adolescent brains is still poorly understood. In the present study, we used the weight-drop model [8] in adolescent male mice, which induces a controlled closed-head trauma and mimics some symptoms found in humans such as brain edema, astrogliosis and cognitive deficit [1,9]. Moreover, the majority of the animal studies on TBI have focused on immediate effects (1 to 24 h) after injury [10–13]; however much less is known about mid- and long-term effects. Here we show the effects of TBI at different timepoints after injury, including short (24h), early mid-term (72h) and late mid-term (two weeks).

The endocannabinoid system (ECS) participates in the resolution of brain injuries, decreasing vasoconstriction, gliosis, neuroinflammation and excitotoxicity [14] and plays an essential role during critical neurodevelopmental periods such as adolescence [15]. The blockage of cannabinoid receptors (CB1 and CB2) results in more severe sequelae after TBI [16] and prevents the anti-gliotic actions of estradiol [17] and the neuroprotective effects of minocycline [16]. Astrogliosis is commonly assessed by changes in vimentin expression which is an intermediate filament responsible for maintaining astrocyte cell integrity [18]. Vimentin is overexpressed by astrocytes after central nervous system (CNS) injury or in neurodegenerative diseases [19] and its levels are a reliable indicator of reactive astrogliosis in the TBI model [20].

Brain edema is one of the hallmarks of TBI [8]. It occurs due to the rupture of BBB [13,21] and the entrance of water through aquaporin-4 (AQP4) protein, a channel involved in fluid homeostasis which is mainly expressed on the astrocytic end-feet [22–25]. The regulation of brain edema may be one of the neuroprotective mechanisms elicited by CB1 and CB2 by the downregulation of reactive astrogliosis, since AQP4 is present in these glial cells. In humans, genetic variations in AQP4 gene influence the functional outcome of TBI [26]. However, the role of AQP4 in TBI is unclear since AQP4 knockout mice present impairments in the clearance of vasogenic edema after lesion [27] but are neuroprotected against cytotoxic edema [28]. Furthermore, brain AQP4 silencing in rats improves functional recover after TBI [29].

In this study in the brain of adolescent male mice we have determined the time course of the changes in the expression of several molecules known to present early modifications after lesion (CB1, CB2, AQP4 and vimentin) and we have followed their evolution up to two weeks, which could be considered as “late mid-term” effects of TBI. A very important input of this study is the analysis of whether the expression of these molecules correlated with neurological deficit and brain edema.

## Materials and Methods

### Animals

All the experiments were performed in Swiss male mice (Harlan, Spain). All the animals sustained TBI protocol at postnatal day (pnd) 35 and then the mice from different groups were sacrificed at pnd36, pnd 38 and pnd 49 covering pre-, mid- and post- adolescence respectively [30]. The range of weight varied from 28 (pnd35) to 37 g (pnd49). Animals were housed in a controlled temperature environment (22 ± 2°C), 12 h light/dark cycle and with free access to food and water. Animal care and procedures were approved by our institutional animal use and care committee (Comité de Experimentación Animal CEA-UCM; 68/2012) and followed the Spanish regulations (Ley 6/2013, 11^th^ June) and the European Communities Council Directive (2010/63/EU) on the protection of animals for experimental use.

A total of 37 animals were used and 19 of them sustained TBI, two of them died immediately after trauma, which meant a mortality rate of 10.53%. Finally, 18 naïve and 17 TBI animals were included for all the assessments. Animals were sacrificed at 24 h (N = 6), 72 h (N = 5) and two weeks (N = 6) after traumatic brain injury (TBI), what corresponded to pnd 36, pnd 38 and pnd 49 respectively. Naïve animals where sacrificed at these same times (N = 6 for all the groups).

### Body weight control

Animals were weighted 24 h before being subjected to TBI model and once again immediately before the sacrifice in order to characterize their general status and well-being. This parameter is used to describe the severity of the model, taking into account that 5–10% body weight (b.w.) loss is associated with a moderate lesion, 10–20% b.w. loss is associated with a severe lesion and more than 20% b.w. loss represents and endpoint criteria (Directive 2010/63/EU).

### Traumatic brain injury

TBI mouse model was performed as previously described [16] at pnd 35, corresponding to the early adolescence period. Prior to the protocol, each animal was randomly assigned to one of the different groups of the study. Mice were anesthetized with 2% isofluorane (IsoFlo, Esteve) before being subjected to TBI. Closed-head trauma was induced by a 50 g weight dropped from a 36 cm height along a stainless steel rod, on the right frontal side of the head. This experimental paradigm creates a limited contra-coup lesion in the right hemisphere (orbitofrontal cortex and perirhinal cortex), accompanied with functional deficit and a 5–15% mortality rate within the first 5 min following the impact [31–33].

### Neurological deficit assessment

The functional outcome was assessed 24, 72 hours and two weeks after TBI by a person that was blind to the experimental groups. This test is a variation of a previous one which considered 10 essential parameters easy to evaluate, objective in interpretation and independent to the subjective evaluation of the researcher [34,35]. The test was conducted in an open circular plastic arena (16 cm height and 30 cm diameter) illuminated 50–50% that contained an exit aperture (2 × 2.5 cm) located in the brighter area. The animal was initially placed in the darker zone and was allowed to explore freely for 2 min. Table 1 resumes the score marks for this test.

**Table 1:** Neurological Score test for mice. Circle exit task and physiologic parameters.

**Task**	**Description**	**Points**
Circle exit	Exit the device < 2min. The animal performed risk evaluation behaviors (head-dipping or stretched attend posture).	3 points
	Exit the device > 2min. The animal performed risk evaluation behaviors (head-dipping or stretched attend posture).	2 points
	Exit the device < 2 min. The animal did not perform risk evaluation behaviors (head-dipping or stretched attend posture).	1 point
	No exit. The animal did not perform risk evaluation behaviors (head-dipping or stretched attend posture).	0 points
**Parameter**	**Description**	**Yes / No**
Alertness	Reaction to stimuli, vigilance in the cage. Eyes and ears alert.	1 / 0
Posture	Four paws on the cage, normal coat appearance, no pain signs (hunched, piloerection).	1 / 0
Exploration	Rearing onto hind legs and sniffing.	1 / 0
Blepharoptosis	Falling of the upper or lower eyelid.	0 / 1
Stereotypes	Repetitive or maladaptive behaviors.	0 / 1

Based on [33,71].

Regarding neurological score test, we split the animals in two groups: High deficit and Low deficit. We set ≤ 5 as High and ≥ 6 as Low deficit because 5 is the minimum mark animals can reach even if they do not exit the circle. After this criterion, 11 mice were included in “High deficit” and 24 in “Low deficit”.

### Cerebral edema evaluation

Cerebral edema was evaluated in the left hemisphere, contralateral to the lesion since the right hemisphere was used for PCR and Western blot analyses. Previous studies have shown that BBB breakdown is also increased in the contralateral hemisphere at 24 h after lesion and that water content in the contralateral hemisphere is a reliable indicator of edema formation [36]. Measurement of the brain water content (BWC) was performed as previously described [31,33,37]. Briefly, animals were sacrificed at 24 h, 72 h and two weeks after TBI by cervical dislocation and the brain was gently removed. A region of tissue (75–100 mg) from the left hemisphere (3–0 mm from bregma) was punched-out with a cannula of 5 mm inner diameter and immediately weighed in order to obtain the wet weight (WW) and heated at 100°C for 24 h. Then, samples were weighed again to obtain the dry weight (DW). BWC was calculated as follows: % H_2_O = [(WW − DW)/ WW] × 100.

### Tissue homogenization and RNA and protein extraction

Animals were sacrificed at 24 h, 72h and two weeks after TBI by cervical dislocation and the brain was gently removed. A region of tissue (75–100 mg) from the right hemisphere (3–0 mm from bregma), ipsilateral to the lesion, was punched-out with a cannula of 5 mm inner diameter and immediately frozen at -80°C. RNA and protein were obtained by double extraction protocol with Trizol reagent (TRI Reagent Solution, Ambion) according to the manufacturer’s instructions. We proceeded to the phase separation, keeping the phenol phase for protein isolation and the aqueous phase for RNA extraction.

### Quantitative Real-Time Polymerase Chain Reaction (qRT-PCR)

After RNA isolation, first-strand cDNA was prepared from 2 μg RNA using M-MLV reverse transcriptase (Promega, Madison, WI, USA) according to the manufacturer’s protocol. After reverse transcription, cDNA was diluted 1:3 for cannabinoid receptor 2 (CB2); 1:8 for cannabinoid receptor 1 (CB1); 1:20 for aquaporin-4 (AQP4); 1:100 for vimentin and 1:300 for the housekeeping gene (18S). 5 μl of these cDNA solutions were amplified by real-time PCR in 15 μl volume reaction using SYBR Green Master Mix (Applied Biosystems, Foster City, CA, USA) using the ABI Prism 7500 Sequence Detection System (Applied Biosystems) with conventional Applied Biosystems cycling parameters (40 cycles of changing temperatures, first at 95°C for 15 s and then 60°C for a minute). All the primer sequences were designed using Primer Express software (Applied Biosystems) and are shown in Table 2.

**Table 2:** Primer sequences for quantitative real-time polymerase chain reaction.

Gene	Forward primer	Reverse primer
CB1	5’-TGCTGGTGCTATGTGTCATCCT-3’	5’-CAAAGCTGTAGACAAAGATGACACTTC-3’
CB2	5’-TGGTCACCACGCTGAGTGA-3’	5’-CCGCAGGGCGTAAATGATAG-3’
AQP4	5’-CCTGATGTGGAGCTCAAACGT-3’	5’-CCACTTGGCTCCGGTTGT-3’
Vimentin	5’-GCTGCAGGCCCAGATTCA-3’	5’-TTCATACTGCTGGCGCACAT-3’
18S	5’-CGCCGCTAGAGGTGAAATTCT-3’	5’-CATTCTTGGCAAATGTCTTTCG-3’

### Western blot

After protein isolation, the samples were boiled for 5 min. Solubilized proteins (30 μg) were resolved by 10% SDS–PAGE at 100 V at room temperature and then transferred to 0.2 μm nitrocellulose membranes (Trans-Blot, Bio-Rad) by a semi-dry system 25 V, 1.0 A, 30 min (Trans-Blot Turbo Transfer System, Bio-Rad). The membranes were treated with 5% (w/v) BSA in TTBS (138 mM NaCl, 25 mM Tris, pH 8.0, and 0.1% (w/v) Tween-20) at room temperature for 3 h, and then incubated overnight at 4°C with the primary antibody diluted in this same blocking solution (see Table 3 for the concentrations of the antibodies). Then, membranes were incubated with the secondary antibody diluted in TTBS for 1.5 h at room temperature. Antibody reaction was visualized by ECL chemiluminescence (Amersham). Densitometric analyses were performed by Quantity One Bio-Rad software and data were normalized to β-actin as protein control and represented as percentage relative to Naïve 24h group.

**Table 3:** Primary antibodies and dilutions used for Western Blot analyses.

Primary Antibody	Host	Dilution	Supplier
CB1 receptor	Rabbit Polyclonal	1:1000	Frontier Institute CB1-Rb-Af380-1
CB2 receptor	Goat Polyclonal	1:1000	Santa Cruz Sc-10076
AQP-4	Rabbit Polyclonal	1:1000	Sigma HPA014784
Vimentin	Rabbit Polyclonal	1:1000	Sigma HPA001762
β-actin	Mouse Monoclonal	1:4000	Sigma AC-74

### Statistical Analysis

Data were analyzed using a two-way analysis of variance (ANOVA), with factors being treatment (TBI or naïve) and time (24, 72 hours and two weeks). Data were not always normally distributed. Therefore, to satisfy the assumption of normality for the ANOVA, we transformed the data when necessary by the natural logarithm function. If transformed data were not normally distributed, nonparametric tests were used (Kruskal–Wallis and post hoc pair-wise comparisons with Mann–Whitney U-test). When appropriate, two-way ANOVAs were followed by separate one-way ANOVA split by the independent factors to further analyze the data. Post hoc comparisons were performed with a level of significance set at p <0.05. For data that were normally distributed and homoscedastic, we used a standard parametric post hoc test (Bonferroni’s test) and for those that were normally distributed, but nonhomoscedastic, we performed nonparametric post hoc comparisons (Games–Howell’s test). Student’s t-test was used when two-group comparison was necessary. Data from all the groups were pooled and Spearman’s rho was used to identify bivariant correlations followed by linear regression test. Statistical analyses were carried out with the SPSS 19.0 software package (SPSS, Inc., Chicago, IL, USA). Data are presented as mean + standard error of the mean (SEM).

## Results

In this study we analyzed the time course of the changes in the endocannabinoid system (CB1 and CB2 receptors), BBB proteins (AQP4) and neuroinflammation markers (vimentin) in adolescent male mice after TBI. Furthermore, we have analyzed the possible associations of these molecules with neurological deficit and brain edema.

### Posttraumatic survival

Initially, we used a total of 45 males and 20 out of them sustained TBI. Within 5 min following TBI, adolescent male mice showed a mortality rate of 10.53%. This acute mortality is comparable to previously published percentages with this weight drop model [31,32,38].

### Body weight

**Fig 1:**
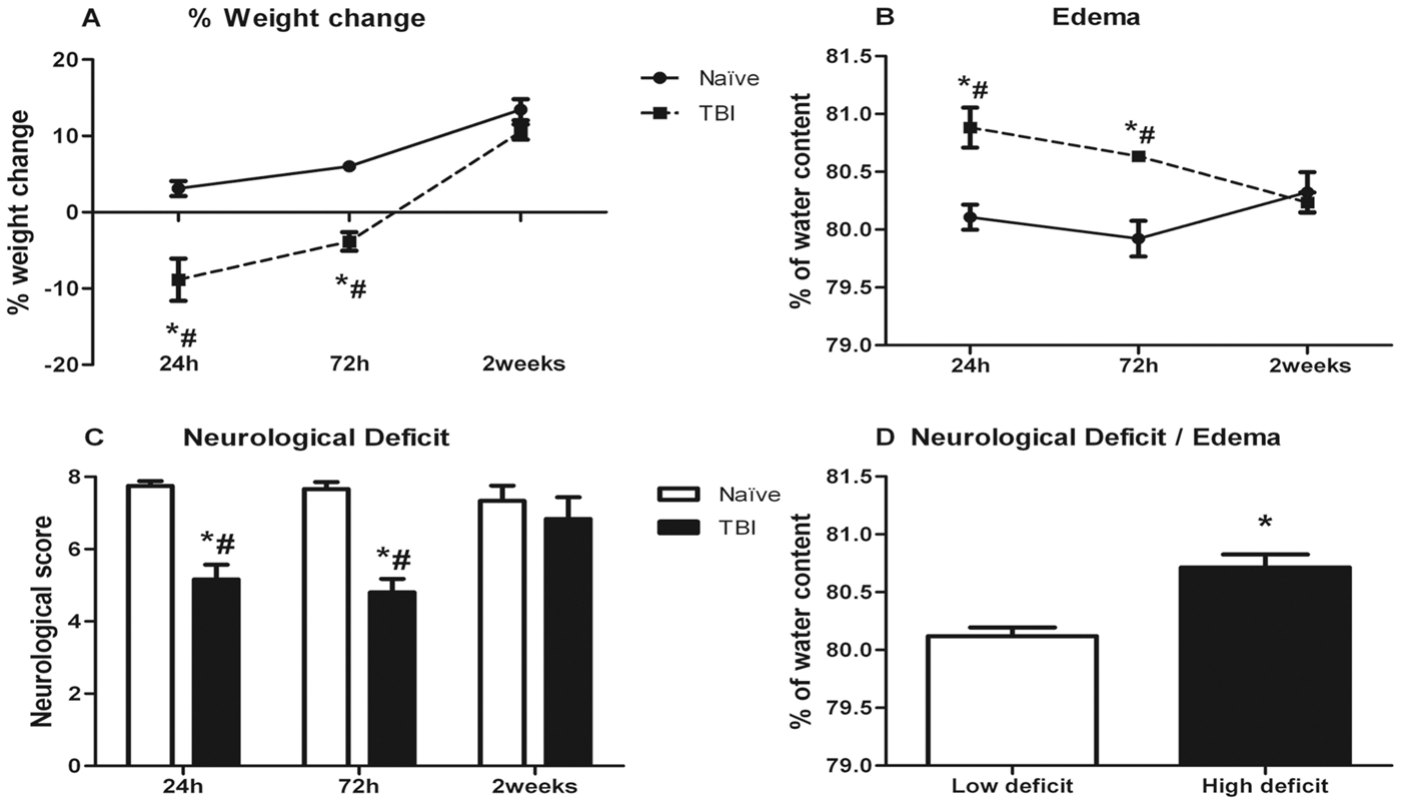
Effects of TBI on body weight, brain edema and neurological deficit. A) Percentage of b.w. change at 24 h, 72 h and two weeks after TBI. B) Brain edema. C) Neurological score. D) Brain edema in animals classified according to neurological score. Data are mean±SEM. * p< 0.05 versus naïve group of same time; # p<0.05 versus two weeks of same treatment.

The percentage of b.w. change after TBI is represented in Fig 1A. Naïve males showed a normal increase in b.w. with age. TBI resulted in a significant decrease of b.w. at 24 and 72 h compared to naïve mice although they also showed a progressive b.w. change with age. Two way ANOVA showed significant effect of treatment [F(1,41) = 57.484] and time [F(2,41) = 63.880] and a significant treatment*time interaction [F(2,41) = 6.004]. Post-hoc comparisons revealed a decrease of the percentage of b.w. change compared to their controls at 24 h (p<0.0001) and 72 h (p = 0.004) after TBI. Injured animals at two weeks after trauma significantly differed from those at 24 h (p<0.0001) and 72 h (p = 0.008) after TBI.

### Brain edema

Brain water content is represented in Fig 1B. Brain edema increased at 24 and 72 h after TBI and recovered to naïve levels by two weeks after lesion. Two way ANOVA showed a significant effect of treatment [F(1,41) = 13.692] and a significant treatment*time interaction [F(2,41) = 4.750]. Subsequent one way ANOVA split by treatment revealed a significant effect of time in injured animals [F(2,14) = 7.685]. Post hoc comparisons showed a significant increase in brain edema at 24 h (p = 0.007) and 72 h (p = 0.030) after TBI.

### Neurological Score

Neurological score is represented in Fig 1C. Neurological score was reduced at 24 and 72 h after TBI and recovered to control values by two weeks. Neurological score data were not normally distributed and therefore data were analyzed using the non-parametric Kruskal-Wallis test. Data was split by time, revealing that TBI induced a significant decrease in neurological score at 24 h (p<0.0001) and 72 h (p = 0.001) after lesion that was significantly recovered at two weeks after trauma.

### Brain edema/Neurological Score

Edema data were split in “High/Low” deficit according to the neurological score. “High deficit” corresponded to low neurological score marks and “Low deficit” to high marks (Fig 1D). Student’s t-test revealed that animals with high deficit show higher brain edema values (p<0.0001).

### CB1 receptor

**Fig 2:**
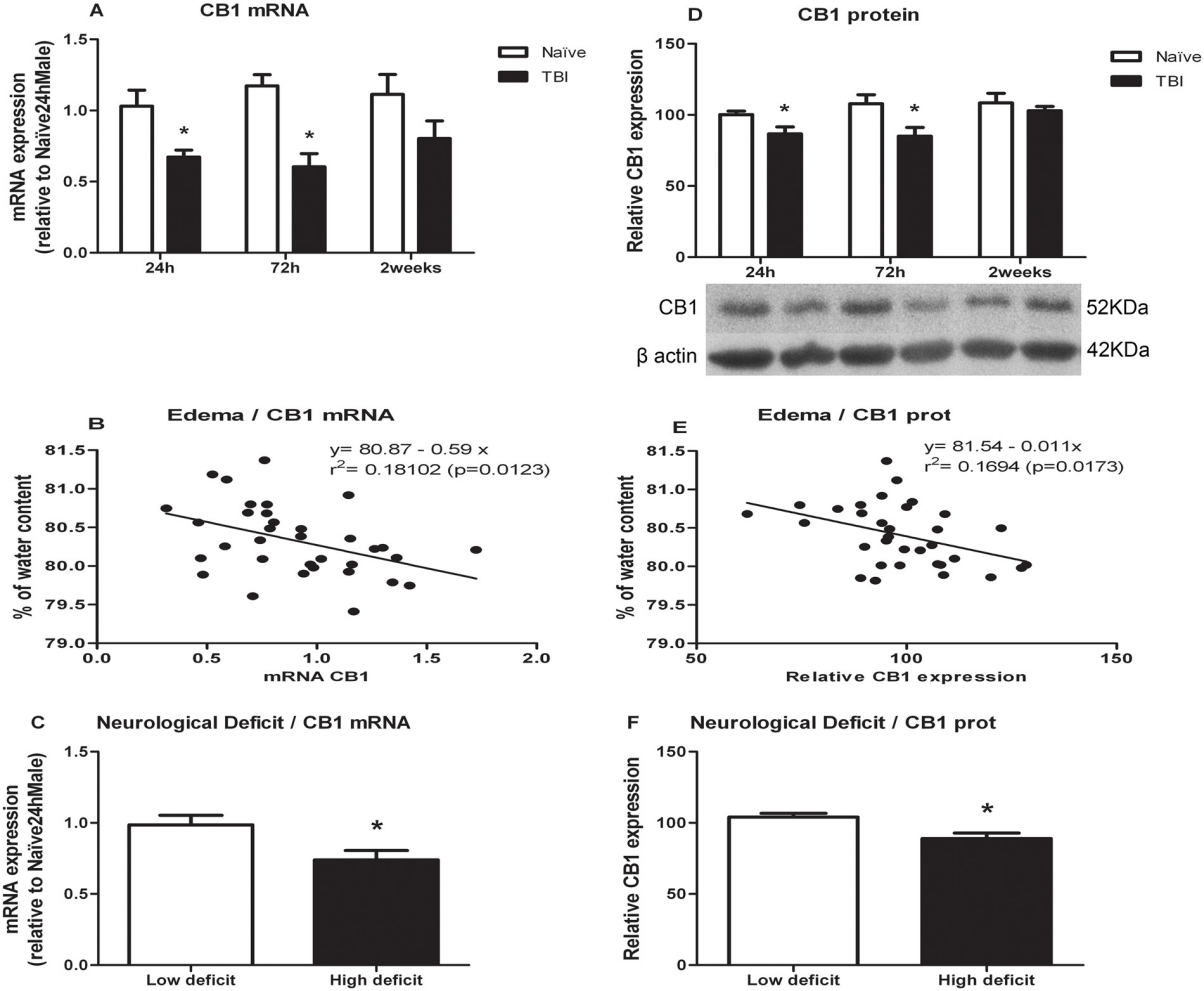
Effects of TBI on CB1 mRNA and protein levels. A) CB1 mRNA levels. B) Analysis of correlation between brain edema and CB1 mRNA levels. C) CB1 mRNA levels in animals classified according to neurological score. D) CB1 protein levels. E) Analysis of correlation between brain edema and CB1 protein levels. F) CB1 protein levels in animals classified according to neurological score. Data are mean±SEM. * p< 0.05 versus naïve group of same time.

Data for CB1 receptor changes are represented in Fig 2. CB1 mRNA and protein levels were significantly decreased 24 and 72 h after TBI and recovered to control values by two weeks. Regarding mRNA levels (Fig 2A), two way ANOVA revealed a significant effect of treatment [F(1,29) = 23.258]. One way ANOVA split by time, showed a significant effect of treatment at 24 [F(1,10) = 8.672; p = 0.015] and 72 hours [F(1,9) = 21.973; p = 0.001]. For protein (Fig 2D), two way ANOVA showed a significant effect of treatment [F(1,27) = 11.172]. One way ANOVA split by time, revealed a significant effect of treatment at 24 [F(1,9) = 6.862; p = 0.028] and 72 hours [F(1,8) = 6.672; p = 0.032].

Pearson’s test showed a significant association of brain edema and CB1 mRNA levels (p = 0.012; r = -0.424) with a negative correlation (r^2^ = 0.181, n = 34, p = 0.012). For CB1 protein, Pearson’s test indicated a significant association (p = 0.011; r = -0.444) with a negative correlation (r^2^ = 0.169, n = 32, p = 0.017) (Fig 2B and 2E, respectively).

Data for CB1 mRNA and protein levels were split in “High/Low” deficit according to the neurological score (Fig 2C and 2F). Student-t test showed that animals with higher deficit, presented lower CB1 mRNA levels (p = 0.032) (Fig 2C). Also for protein, mice with higher deficit expressed lower CB1 receptor (p = 0.024) (Fig 2F).

### CB2 receptor

**Fig 3:**
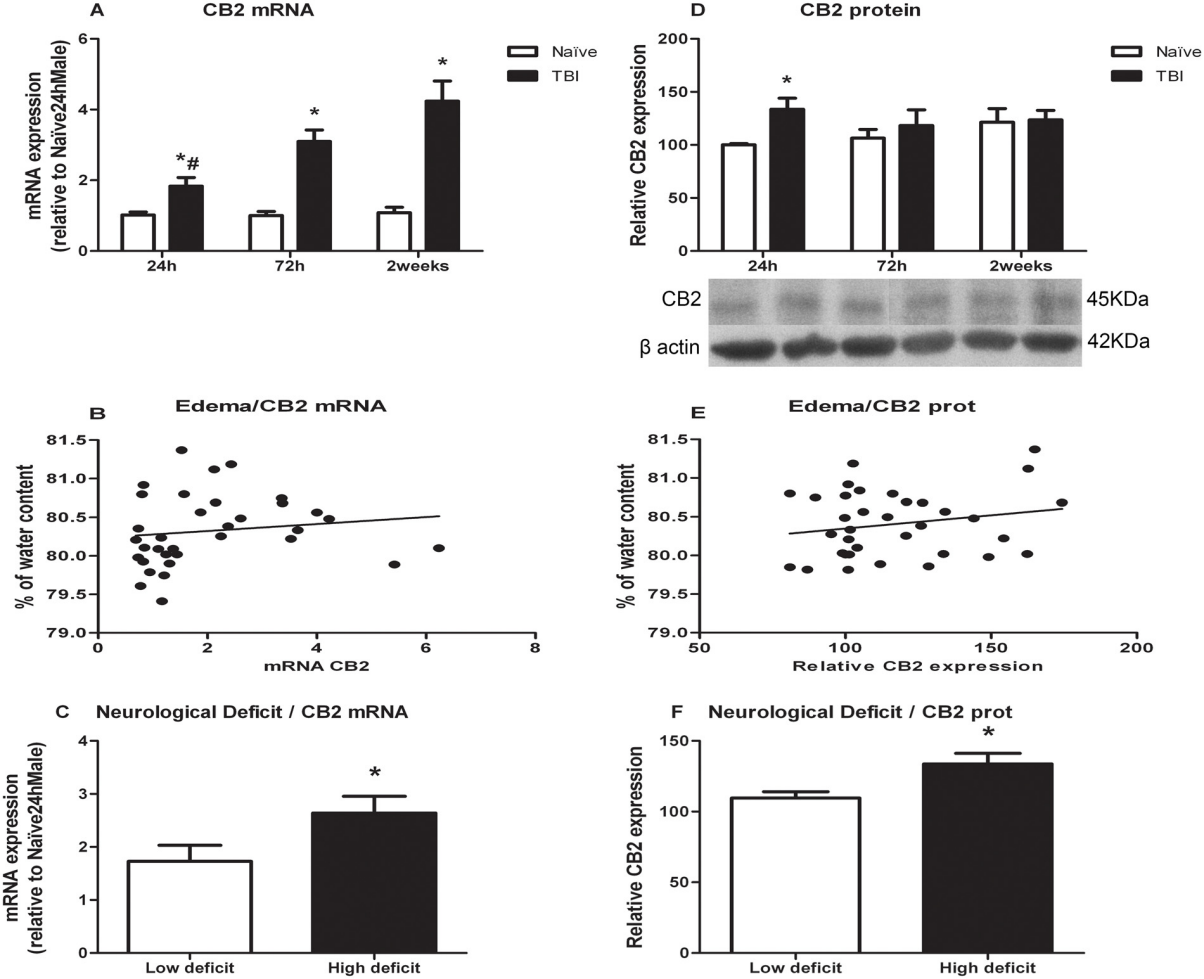
Effects of TBI on CB2 mRNA and protein levels. A) CB2 mRNA levels. B) Analysis of correlation between brain edema and CB2 mRNA levels. C) CB2 mRNA levels in animals classified according to neurological score. D) CB2 protein levels. E) Analysis of correlation between brain edema and CB2 protein levels. F) CB2 protein levels in animals classified according to neurological score. Data are mean±SEM. * p< 0.05 versus naïve group of same time; # p<0.05 versus two weeks of same treatment.

Data related to CB2 receptor are represented in Fig 3. CB2 mRNA levels increased progressively after TBI whereas protein levels only increased 24 h after injury. For mRNA levels (Fig 3A), two way ANOVA showed a significant effect of treatment [F(1,29) = 89.259], time [F(2,29) = 5.763] and a significant treatment*time interaction [F(2,29) = 5.325]. One way ANOVA split by time, revealed a significant effect of treatment at 24 h [F(1,10) = 8.596; p = 0.015], 72 h [F(1,9) = 48.329; p<0.0001] and two weeks [F(1,10) = 45.548; p<0.0001]. For proteins (Fig 3D), two way ANOVA revealed a significant effect of treatment [F(1,29) = 5.319]. Post-hoc analyses confirmed an increase in CB2 protein expression at 24 h after TBI compared to control values (p = 0.015).

Spearman’s test did not reveal a significant association of brain edema and CB2 mRNA or protein levels (Fig 3B and 3E).

Data for CB2 mRNA and protein levels were split in “High/Low” deficit according to the neurological score (Fig 3C and 3F,). Student’s t-test revealed that mice with high neurological deficit expressed high CB2 mRNA levels (p = 0.013) and high protein expression (p = 0.011) (Fig 3C and 3F, respectively).

### Aquaporin-4

**Fig 4:**
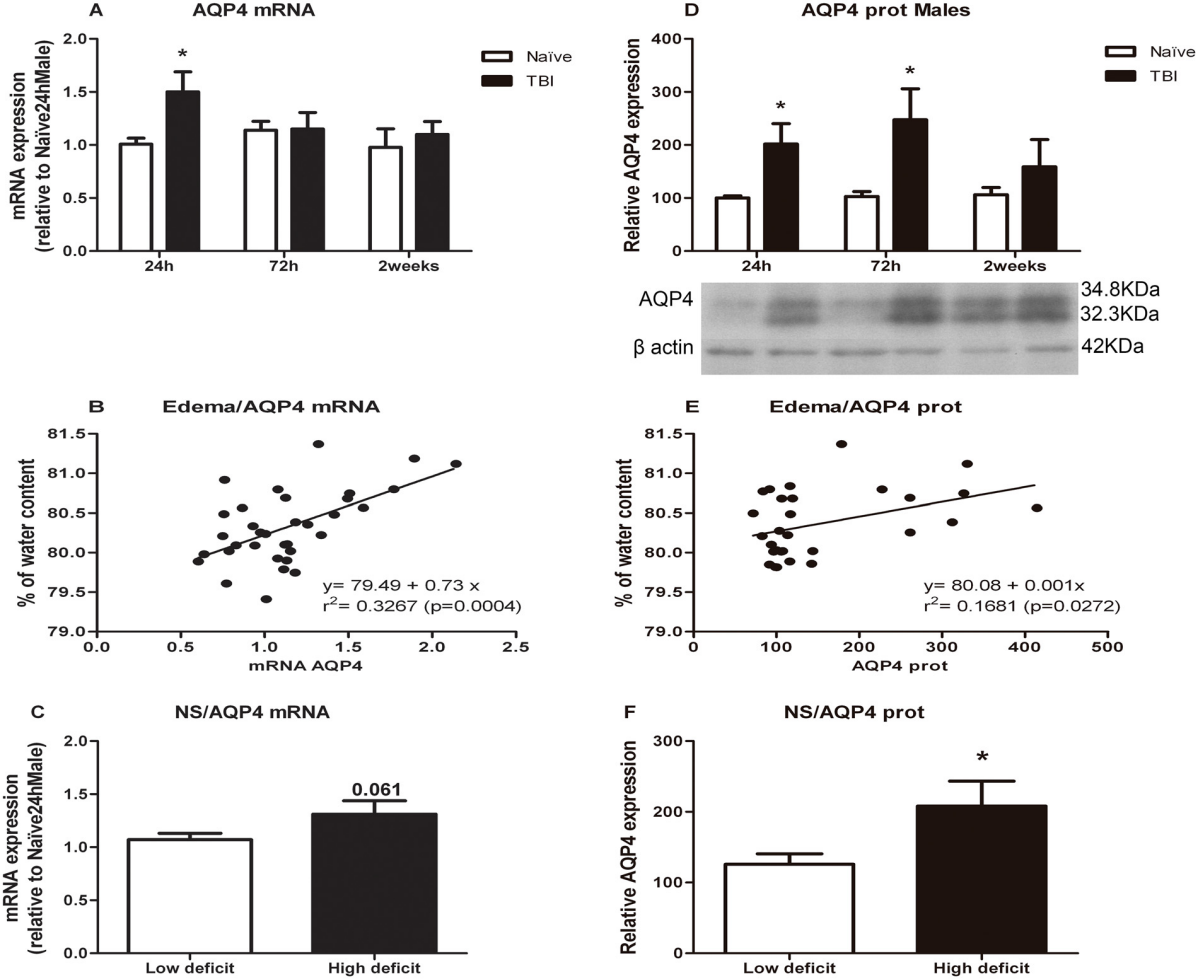
Effects of TBI on AQP4 mRNA and protein levels. A) AQP4 mRNA levels. B) Analysis of correlation between brain edema and AQP4 mRNA levels. C) AQP4 mRNA levels in animals classified according to neurological score. D) AQP4 protein levels. E) Analysis of correlation between brain edema and AQP4 protein levels. F) AQP4 protein levels in animals classified according to neurological score. Data are mean±SEM. * p< 0.05 versus naïve group of same time.

Results for AQP4 are represented in Fig 4. AQP4 mRNA levels were significantly increased 24 h after whereas protein expression increased at 24 h and 72 h after injury.

For mRNA levels (Fig 4A), one way ANOVA split by time, revealed a significant effect of treatment at 24 h after lesion [F(1,10) = 6.332; p = 0.031]. In protein (Fig 4D), two way ANOVA showed a significant effect of the treatment [F(1,25) = 13.894]. One way ANOVA split by time, revealed a significant effect of treatment 24 h [F(1,10) = 5.752; p = 0.037] and 72 h [F(1,9) = 14.579; p = 0.004] after TBI.

Pearson’s test revealed a significant association of brain edema and AQP4 mRNA levels (p<0.0001; r = 0.572) with a positive correlation (r^2^ = 0.326, n = 34, p = 0.0004). For protein, Spearman’s test showed a high trend of association (p = 0.053) for this same positive correlation (Fig 4B and 4E, respectively).

Student’s t-test showed that animals with higher neurological deficit showed higher AQP4 protein levels (p = 0.010) (Fig 4F), but no significant difference in AQP4 mRNA levels was found (Fig 4C).

### Vimentin

**Fig 5:**
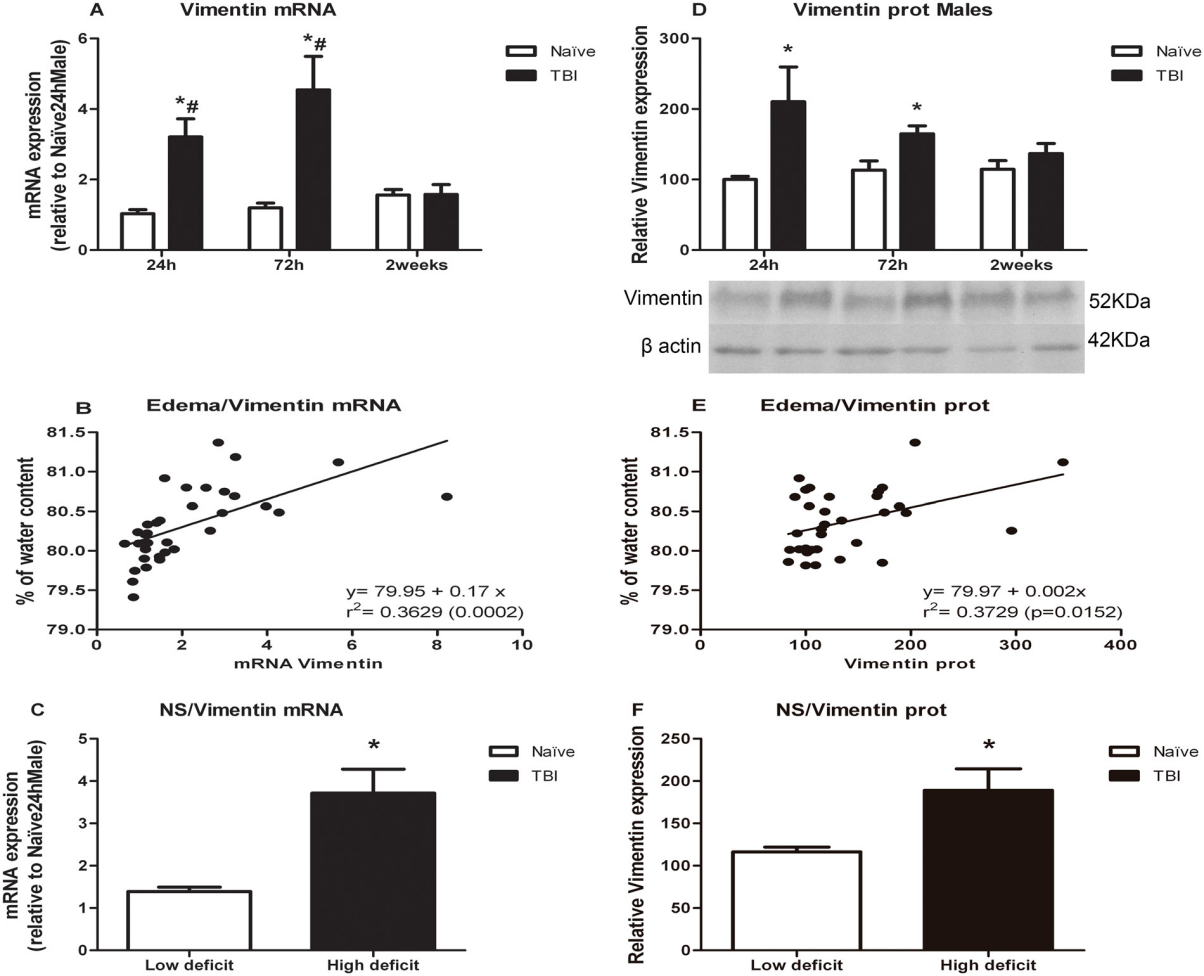
Effects of TBI on vimentin mRNA and protein levels. A) Vimentin mRNA levels. B) Analysis of correlation between brain edema and vimentin mRNA levels. C) Vimentin mRNA levels in animals classified according to neurological score. D) Vimentin protein levels. E) Analysis of correlation between brain edema and vimentin protein levels. F) Vimentin protein levels in animals classified according to neurological score. Data are mean±SEM. * p< 0.05 versus naïve group of same time; # p<0.05 versus two weeks of same treatment.

Vimentin results are represented in Fig 5. Vimentin mRNA and protein levels increased 24 h and 72 h after TBI and recovered to control values by two weeks. In mRNA levels (Fig 5A), two way ANOVA revealed a significant effect of the treatment [F(1,29) = 55.141], time [F(2,29) = 4.346] as well as a significant treatment*time interaction [F(2,29) = 15.053]. Post-hoc comparisons showed a significant increase in vimentin mRNA levels at 24 h (p<0.0001) and 72 h (p<0.0001) after TBI that was significantly recovered by two weeks after injury (p = 0.007 and p<0.0001 for 24 and 72 h respectively). In the case of proteins, two way ANOVA showed a significant effect of treatment [F(1,27) = 15.863]. One way ANOVA split by time revealed a significant effect of treatment in the levels of vimentin at 24 h [F(1,8) = 7.827; p = 0.023] and 72 h [F(1,9) = 7.407; p = 0.024].

Spearman’s test revealed a significant association of brain edema with vimentin mRNA levels (p<0.0001; r = 0.760) with a positive correlation (r^2^ = 0.362, n = 34, p = 0.0002). For protein, Spearman’s test showed a significant association (p = 0.045; r = 0.357) with a positive correlation (r^2^ = 0.179, n = 32, p = 0.0156) (Fig 5B and 5E, respectively).

Student’s t-test showed that vimentin mRNA (p<0.0001) and protein (p = 0.003) levels were higher in animals with high neurological deficit (Fig 5C and 5F, respectively).

## Discussion

In this study we have analyzed the possible contribution of the changes of the endocannabinoid system (CB1 and CB2 receptors), BBB proteins (AQP4) and neuroinflammation markers (vimentin) to neurological deficit and brain edema after TBI in adolescent male mice. Previous studies have shown that the expression of these molecules is altered shortly after TBI [16,27,39–41]. Our present findings suggest that these changes influence the functional recovery after injury.

The pathophysiology of TBI is very variable and complex and it is poorly understood in critical neurodevelopmental periods such as adolescence. The experimental models used to study the impact of TBI at this age have used cortical ablation [42], scaled cortical impact [43] or exposed the dura [44]; however, the highest percentage of TBI patients present close-head trauma injuries [6,7] and so, close-head trauma models such as weight drop model are closer to the clinical reality.

In our hands, weight-drop model generated a moderate lesion characterized by a b.w. loss varying from 10–14% and a mortality rate of 10.53%. Brain edema, one of the hallmarks of TBI caused by the disruption of BBB [1], increased 24 and 72 h after TBI and disappeared two weeks after lesion which agrees with previous studies showing that brain edema increases up to five days after lesion [10] and completely disappears after two weeks [45].

In humans, TBI induces impulsive behavior [46] and deficit in spatial learning and memory [47]. As we have previously described in mice, TBI causes neurological impairments 24 h after lesion [16]. Here we confirm this previous result and also show that deficit lasted up to 72 h after TBI and disappeared two weeks after trauma. Neurological impairments were associated with high levels of brain edema which is in line with previous studies showing that behavioral alterations correlate with cell damage that in turn is directly related to brain edema [47]. Furthermore, studies with patients suffering from subarachnoid hemorrhage accompanied by brain swelling found that edema is consistently associated with cognitive impairments, affecting four of the eight cognition domains analyzed [48].

The ECS participates in TBI sequelae decreasing harmful pathways and promoting the resolution of the injury through CB1 and CB2 receptors [14]. CB1 receptor levels decreased after lesion. This decrease could exacerbate the neurological deficit, since animals with high neurological impairments showed lower CB1 levels. In agreement with this observation, CB1 KO mice [49] and animals treated with a CB1 receptor antagonist [16] showed an impaired recovery after trauma that affected edema and neurological score. This finding is related to the key role of CB1 receptor in anxiety and depression-like behaviors [50] and emotional homeostasis [51] which would also affect the neurological score test performance. CB1 expression presented a negative correlation with brain edema. Since CB1 is located in the end-foot of astrocytes [52], which are the principal glial cells that control ion exchange [53] and modulate cerebral blood flow [54], a lower expression of CB1 could affect the ionic balance and may lead to altered brain edema formation and resolution. Moreover, previous studies [55,56] with endothelium derived from human brain capillaries and microvessels demonstrated that one of the endogenous agonists of CB1 receptor, 2-arachydonoil glycerol (2-AG), inhibits some of the effects of endothelin-1 (ET-1). ET-1 is a potent vasoconstrictor that regulates the responses of brain capillaries and microvessels [55,56] and controls the rearrangement of cytoskeleton (actin and vimentin filaments). The use of selective antagonists of CB1 prevented the effects of 2-AG on ET-1, suggesting that the vasorelaxant function of 2-AG is mediated by CB1 receptor and involves the control of endothelial molecules such as ET-1. As the endothelium plays a key role in the control of vasculature tone and blood flow, which is directly related to edema formation and resolution [1], this suggests that the control of edema through CB1 receptor is also related to the endothelial factor ET-1.

In physiological conditions, CB2 is expressed at very low levels, predominantly in non-neuronal cells [14], although it is also present in neural progenitors, neurons and endothelial cells [57,58]. However, CB2 expression increases under neuroinflammation [59] as observed in our TBI model. High CB2 levels were associated to high neurological impairments, perhaps triggered as a rescue mechanism to reduce brain damage since its pharmacological blockage worsens behavioral deficit after TBI [16]. Moreover, CB2 is commonly related to neuroprotective effects like BBB repair [60] or microglia activation [61] and CB2 agonists induce a better recovery after lesion in behavioral tests [62]. No correlation was found between CB2 and edema, possibly because after TBI this receptor is more expressed in microglia than in astrocytes [59], the principal glial cells involved in edema control. CB1 and CB2 receptors frequently present divergent changes in expression under pathological conditions. For example CB1 receptor expression is decreased, while CB2 expression is increased in glioblastoma multiforme tissue, even if both receptors present the same GTPase activity [63]. We have also demonstrated that the pharmacological blockage of CB2 is more potent than the blockage of CB1 in decreasing the protective actions of estradiol [17] or minocycline [16] in brain lesions. Here we show that CB1 expression decreased and CB2 expression increased after TBI. CB1 receptor is mainly expressed in astrocytes and neurons whereas CB2 is mostly present in microglia cells [59]. Therefore, the decrease in CB1 levels could be the result of the neuronal loss caused by TBI, while the increase in CB2 levels could be associated with the increased microglia activation and proliferation.

AQP4 changes during CNS injury depend on the type of edema generated [27]. Weight-drop models predominantly induce vasogenic edema [64,65], whose resolution is AQP4-dependent [28]. After TBI, AQP4 levels increased up to 72 h after lesion in the case of protein expression, supporting previous studies with weight-drop models [66]. High AQP4 levels were associated with high neurological deficit and presented a positive correlation with brain edema. This suggests that increased AQP4 expression after TBI contributes to increase brain edema, which in turn correlated with neurological impairments. Also, this leads to the study of new approaches to reduce the cognitive and behavioral impairments after TBI by controlling the expression or modulating the activity of AQP4.

After CNS injury astrocytes overexpress vimentin [19] and together with GFAP, is one of the greatest increases in gene expression experimented at 24 h after brain lesion [41]; here we show that this increase is maintained up to 72 h after injury. Elevated levels of vimentin were associated with high neurological deficit and brain edema. There is a tight relation between neuroinflammation and behavior. Animals experiencing immune activation present what is known as “sickness behavior” [67], characterized by reduced food intake and activity or increased sleep [68]. Regarding edema, high vimentin levels could regulate AQP4 mobility and localization, which controls brain water balance [69]. High levels of vimentin probably reflect an increased astrogliosis and consequently, more hypertrophic astrocytes expressing high AQP4 mRNA after the disruption of BBB [70]. The control of AQP4 mobility and location in reactive astrocytes could be an explanation for the antiedematous action of minocycline, a microglia activation inhibitor [31] that secondarily results in the inhibition of reactive astrogliosis.

In summary, our findings indicate that there is a correlation between brain edema and neurological deficit after TBI in adolescent male mice. The negative correlation of CB1 with brain edema and the fact that animals with high neurological deficit showed reduced CB1 expression suggest that this receptor plays a crucial role in the recovery after TBI by the regulation of brain edema. In contrast, our findings showing that high CB2 expression was associated to high neurological impairments and the absence of correlation with edema suggest that CB2 has a different function than CB1 after TBI; possibly the expression of CB2 increases as a compensatory or rescue mechanism whereas CB1 expression is decreased possibly due to neuronal loss. The regulation of astrogliosis and AQP4 seems to be critical for the outcome of TBI as well. Thus, higher expression of AQP4 and vimentin were associated with high neurological deficit and showed a positive correlation with brain edema. These findings suggest that the reported role of CB1 after CNS injury in the control of astrogliosis, assessed by vimentin expression [17], may contribute to CB1-mediated neuroprotection by the regulation of AQP4 levels in reactive astrocytes.

## References

[1] Chodobski A, Zink BJ, Szmydynger-Chodobska J. Blood-brain barrier pathophysiology in traumatic brain injury. Transl Stroke Res. 2011;2: 492–516. 10.1007/s12975-011-0125-x 22299022 PMC3268209

[2] Bruns J, Hauser WA. The epidemiology of traumatic brain injury: a review. Epilepsia. 2003;44 Suppl 1: 2–10. 10.1046/j.1528-1157.44.s10.3.x 14511388

[3] Spear LP. The adolescent brain and age-related behavioral manifestations. Neurosci Biobehav Rev. 2000;24: 417–63. 10817843 10.1016/s0149-7634(00)00014-2

[4] Adriani W, Chiarotti F, Laviola G. Elevated novelty seeking and peculiar d-amphetamine sensitization in periadolescent mice compared with adult mice. Behav Neurosci. 1998;112: 1152–66. 9829793 10.1037//0735-7044.112.5.1152

[5] Adriani W, Laviola G. Elevated levels of impulsivity and reduced place conditioning with d-amphetamine: two behavioral features of adolescence in mice. Behav Neurosci. 2003;117: 695–703. 12931955 10.1037/0735-7044.117.4.695

[6] Masson F, Thicoipe M, Aye P, Mokni T, Senjean P, Schmitt V, et al Epidemiology of severe brain injuries: a prospective population-based study. J Trauma. 2001;51: 481–9. 11535895 10.1097/00005373-200109000-00010

[7] Wu X, Hu J, Zhuo L, Fu C, Hui G, Wang Y, et al Epidemiology of traumatic brain injury in eastern China, 2004: a prospective large case study. J Trauma. 2008;64: 1313–9. 10.1097/TA.0b013e318165c803 18469656

[8] Marmarou A. Pathophysiology of traumatic brain edema: current concepts. Acta Neurochir Suppl. 2003;86: 7–10. 14753394 10.1007/978-3-7091-0651-8_2

[9] Siopi E, Cho AH, Homsi S, Croci N, Plotkine M, Marchand-Leroux C, et al Minocycline restores sAPPα levels and reduces the late histopathological consequences of traumatic brain injury in mice. J Neurotrauma. 2011;28: 2135–43. 10.1089/neu.2010.1738 21770756

[10] O’Connor CA, Cernak I, Vink R. The temporal profile of edema formation differs between male and female rats following diffuse traumatic brain injury. Acta Neurochir Suppl. 2006;96: 121–4. 16671438 10.1007/3-211-30714-1_27

[11] Schwarzmaier SM, Zimmermann R, McGarry NB, Trabold R, Kim S-W, Plesnila N. In vivo temporal and spatial profile of leukocyte adhesion and migration after experimental traumatic brain injury in mice. J Neuroinflammation. 2013;10: 32 10.1186/1742-2094-10-32 23448240 PMC3610295

[12] Schwarzmaier SM, Kim S-W, Trabold R, Plesnila N. Temporal profile of thrombogenesis in the cerebral microcirculation after traumatic brain injury in mice. J Neurotrauma. 2010;27: 121–30. 10.1089/neu.2009.1114 19803784

[13] Panikashvili D, Simeonidou C, Ben-Shabat S, Hanus L, Breuer A, Mechoulam R, et al An endogenous cannabinoid (2-AG) is neuroprotective after brain injury. Nature. 2001;413: 527–531. 11586361 10.1038/35097089

[14] Shohami E, Cohen-Yeshurun A, Magid L, Algali M, Mechoulam R. Endocannabinoids and traumatic brain injury. Br J Pharmacol. 2011;163: 1402–10. 10.1111/j.1476-5381.2011.01343.x 21418185 PMC3165950

[15] Viveros MP, Llorente R, Suarez J, Llorente-Berzal A, López-Gallardo M, de Fonseca FR. The endocannabinoid system in critical neurodevelopmental periods: sex differences and neuropsychiatric implications. J Psychopharmacol. 2012;26: 164–76. 10.1177/0269881111408956 21669929

[16] Lopez-Rodriguez AB, Siopi E, Finn DP, Marchand-Leroux C, Garcia-Segura LM, Jafarian-Tehrani M, et al CB1 and CB2 Cannabinoid Receptor Antagonists Prevent Minocycline-Induced Neuroprotection Following Traumatic Brain Injury in Mice. Cereb Cortex. 2015; 1: 35–45. 10.1093/cercor/bht202 23960212

[17] López Rodríguez AB, Mateos Vicente B, Romero-Zerbo SY, Rodriguez-Rodriguez N, Bellini MJ, Rodriguez de Fonseca F, et al Estradiol decreases cortical reactive astrogliosis after brain injury by a mechanism involving cannabinoid receptors. Cereb Cortex. 2011;21: 2046–55. 10.1093/cercor/bhq277 21258044

[18] Franke WW, Grund C, Kuhn C, Jackson BW, Illmensee K. Formation of cytoskeletal elements during mouse embryogenesis. III. Primary mesenchymal cells and the first appearance of vimentin filaments. Differentiation. 1982;23: 43–59. 6759279 10.1111/j.1432-0436.1982.tb01266.x

[19] Pekny M, Wilhelmsson U, Bogestål YR, Pekna M. The role of astrocytes and complement system in neural plasticity. Int Rev Neurobiol. 2007;82: 95–111. 17678957 10.1016/S0074-7742(07)82005-8

[20] Ekmark-Lewén S, Lewén A, Israelsson C, Li GL, Farooque M, Olsson Y, et al Vimentin and GFAP responses in astrocytes after contusion trauma to the murine brain. Restor Neurol Neurosci. 2010;28: 311–21. 10.3233/RNN-2010-0529 20479526

[21] Panikashvili D, Shein NA, Mechoulam R, Trembovler V, Kohen R, Alexandrovich A, et al The endocannabinoid 2-AG protects the blood-brain barrier after closed head injury and inhibits mRNA expression of proinflammatory cytokines. Neurobiol Dis. 2006;22: 257–64. 16364651 10.1016/j.nbd.2005.11.004

[22] Ropper AH, Shafran B. Brain edema after stroke. Clinical syndrome and intracranial pressure. Arch Neurol. 1984;41: 26–9. 6606414 10.1001/archneur.1984.04050130032017

[23] Badaut J, Lasbennes F, Magistretti PJ, Regli L. Aquaporins in brain: distribution, physiology, and pathophysiology. J Cereb Blood Flow Metab. 2002;22: 367–78. 11919508 10.1097/00004647-200204000-00001

[24] Bardutzky J, Schwab S. Antiedema therapy in ischemic stroke. Stroke. 2007;38: 3084–94. 17901384 10.1161/STROKEAHA.107.490193

[25] Ren Z, Iliff JJ, Yang L, Yang J, Chen X, Chen MJ, et al “Hit & Run” model of closed-skull traumatic brain injury (TBI) reveals complex patterns of post-traumatic AQP4 dysregulation. J Cereb Blood Flow Metab. 2013;33: 834–45. 10.1038/jcbfm.2013.30 23443171 PMC3677112

[26] Dardiotis E, Paterakis K, Tsivgoulis G, Tsintou M, Hadjigeorgiou GF, Dardioti M, et al AQP4 tag single nucleotide polymorphisms in patients with traumatic brain injury. J Neurotrauma. 2014; 23:1920–6. 10.1089/neu.2014.3347 24999750 PMC4238262

[27] Verkman AS, Binder DK, Bloch O, Auguste K, Papadopoulos MC. Three distinct roles of aquaporin-4 in brain function revealed by knockout mice. Biochim Biophys Acta. 2006;1758: 1085–93. 16564496 10.1016/j.bbamem.2006.02.018

[28] Yang B, Zador Z, Verkman AS. Glial cell aquaporin-4 overexpression in transgenic mice accelerates cytotoxic brain swelling. J Biol Chem. 2008;283: 15280–6. 10.1074/jbc.M801425200 18375385 PMC2397463

[29] Fukuda AM, Adami A, Pop V, Bellone JA, Coats JS, Hartman RE, et al Posttraumatic reduction of edema with aquaporin-4 RNA interference improves acute and chronic functional recovery. J Cereb Blood Flow Metab. 2013;33: 1621–32. 10.1038/jcbfm.2013.118 23899928 PMC3790933

[30] Adriani W, Granstrem O, Macri S, Izykenova G, Dambinova S, Laviola G. Behavioral and neurochemical vulnerability during adolescence in mice: studies with nicotine. Neuropsychopharmacology. American College of Neuropsychopharmacology; 2004;29: 869–78. 14666123 10.1038/sj.npp.1300366

[31] Homsi S, Federico F, Croci N, Palmier B, Plotkine M, Marchand-Leroux C, et al Minocycline effects on cerebral edema: relations with inflammatory and oxidative stress markers following traumatic brain injury in mice. Brain Res. Elsevier B.V.; 2009;1291: 122–132. 10.1016/j.brainres.2009.07.031 19631631

[32] Homsi S, Piaggio T, Croci N, Noble F, Plotkine M, Marchand-Leroux C, et al Blockade of acute microglial activation by minocycline promotes neuroprotection and reduces locomotor hyperactivity after closed head injury in mice: a twelve-week follow-up study. J Neurotrauma. 2010;27: 911–921. 10.1089/neu.2009.1223 20166806

[33] Siopi E, Llufriu-Dabén G, Fanucchi F, Plotkine M, Marchand-Leroux C, Jafarian-Tehrani M. Evaluation of late cognitive impairment and anxiety states following traumatic brain injury in mice: the effect of minocycline. Neurosci Lett. 2012;511: 110–5. 10.1016/j.neulet.2012.01.051 22314279

[34] Stahel PF, Shohami E, Younis FM, Kariya K, Otto VI, Lenzlinger PM, et al Experimental closed head injury: analysis of neurological outcome, blood-brain barrier dysfunction, intracranial neutrophil infiltration, and neuronal cell death in mice deficient in genes for pro-inflammatory cytokines. J Cereb Blood Flow Metab. 2000;20: 369–80. 10698075 10.1097/00004647-200002000-00019

[35] Flierl M, Stahel PF, Beauchamp KM, Morgan SJ, Smith WR, Shohami E. Mouse closed head injury model induced by a weight-drop device. Nat Protoc. Nature Publishing Group; 2009;4: 1328–1337. 10.1038/nprot.2009.148 19713954

[36] Lin Y, Pan Y, Wang M, Huang X, Yin Y, Wang Y, et al Blood-brain barrier permeability is positively correlated with cerebral microvascular perfusion in the early fluid percussion-injured brain of the rat. Lab Invest. 2012;92: 1623–34. 10.1038/labinvest.2012.118 22964852

[37] Hellal F, Pruneau D, Palmier B, Faye P, Croci N, Plotkine M, et al Detrimental role of bradykinin B2 receptor in a murine model of diffuse brain injury. J Neurotrauma. 2003;20: 841–51. 14577862 10.1089/089771503322385773

[38] Siopi E, Calabria S, Plotkine M, Marchand-Leroux C, Jafarian-Tehrani M. Minocycline restores olfactory bulb volume and olfactory behavior after traumatic brain injury in mice. J Neurotrauma. 2012;29: 354–61. 10.1089/neu.2011.2055 21910642

[39] Shohami E, Cohen-Yeshurun A, Magid L, Algali M, Mechoulam R. Endocannabinoids and traumatic brain injury. Mol Neurobiol. 2007;163: 68–74. 10.1007/s12035-007-8008-6 17952651

[40] Kapoor S, Kim S-M, Farook JM, Mir S, Saha R, Sen N. Foxo3a transcriptionally upregulates AQP4 and induces cerebral edema following traumatic brain injury. J Neurosci. 2013;33: 17398–403. 10.1523/JNEUROSCI.2756-13.2013 24174672 PMC6618370

[41] Kochanek PM, Dixon CE, Shellington DK, Shin SS, Bayır H, Jackson EK, et al Screening of biochemical and molecular mechanisms of secondary injury and repair in the brain after experimental blast-induced traumatic brain injury in rats. J Neurotrauma. 2013;30: 920–37. 10.1089/neu.2013.2862 23496248 PMC5586163

[42] Kolb B, Whishaw IQ, van der Kooy D. Brain development in the neonatally decorticated rat. Brain Res. 1986;397: 315–26. 3801872 10.1016/0006-8993(86)90633-5

[43] Duhaime AC, Margulies SS, Durham SR, O’Rourke MM, Golden JA, Marwaha S, et al Maturation-dependent response of the piglet brain to scaled cortical impact. J Neurosurg. 2000;93: 455–62. 10969944 10.3171/jns.2000.93.3.0455

[44] Adelson PD, Whalen MJ, Kochanek PM, Robichaud P, Carlos TM. Blood brain barrier permeability and acute inflammation in two models of traumatic brain injury in the immature rat: a preliminary report. Acta Neurochir Suppl. 1998;71: 104–6. 9779157 10.1007/978-3-7091-6475-4_31

[45] Hellal F, Bonnefont-Rousselot D, Croci N, Palmier B, Plotkine M, Marchand-Verrecchia C. Pattern of cerebral edema and hemorrhage in a mice model of diffuse brain injury. Neurosci Lett. 2004;357: 21–4. 15036604 10.1016/j.neulet.2003.12.036

[46] Rochat L, Beni C, Billieux J, Azouvi P, Annoni J-M, Van der Linden M. Assessment of impulsivity after moderate to severe traumatic brain injury. Neuropsychol Rehabil. 2010;20: 778–97. 10.1080/09602011.2010.495245 20635306

[47] Shenaq M, Kassem H, Peng C, Schafer S, Ding JY, Fredrickson V, et al Neuronal damage and functional deficits are ameliorated by inhibition of aquaporin and HIF1α after traumatic brain injury (TBI). J Neurol Sci. Elsevier B.V.; 2012;323: 134–40. 10.1016/j.jns.2012.08.036 23040263

[48] Kreiter KT, Copeland D, Bernardini GL, Bates JE, Peery S, Claassen J, et al Predictors of cognitive dysfunction after subarachnoid hemorrhage. Stroke. 2002;33: 200–8. 11779911 10.1161/hs0102.101080

[49] Panikashvili D, Mechoulam R, Beni SM, Alexandrovich A, Shohami E. CB1 cannabinoid receptors are involved in neuroprotection via NF-kappa B inhibition. J Cereb Blood Flow Metab. 2005;25: 477–84. 15729296 10.1038/sj.jcbfm.9600047

[50] Litvin Y, Phan A, Hill MN, Pfaff DW, McEwen BS. CB1 receptor signaling regulates social anxiety and memory. Genes Brain Behav. 2013;12: 479–89. 10.1111/gbb.12045 23647582

[51] Marco EM, Viveros MP. The critical role of the endocannabinoid system in emotional homeostasis: avoiding excess and deficiencies. Mini Rev Med Chem. 2009;9: 1407–15. 19929814 10.2174/138955709789957468

[52] Rodriguez JJ, Mackie K, Pickel VM. Ultrastructural localization of the CB1 cannabinoid receptor in mu-opioid receptor patches of the rat Caudate putamen nucleus. J Neurosci. 2001;21: 823–33. 11157068 10.1523/JNEUROSCI.21-03-00823.2001 PMC6762333

[53] Straiker A, Mackie K. Cannabinoids, electrophysiology, and retrograde messengers: challenges for the next 5 years. AAPS J. 2006;8: E272–6. 16796377 10.1007/BF02854897 PMC3231565

[54] Iring A, Ruisanchez É, Leszl-Ishiguro M, Horváth B, Benkő R, Lacza Z, et al Role of endocannabinoids and cannabinoid-1 receptors in cerebrocortical blood flow regulation. PLoS One. 2013;8: e53390 10.1371/journal.pone.0053390 23308211 PMC3537620

[55] Chen Y, McCarron RM, Ohara Y, Bembry J, Azzam N, Lenz FA, et al Human brain capillary endothelium: 2-arachidonoglycerol (endocannabinoid) interacts with endothelin-1. Circ Res. 2000;87: 323–7. 10948067 10.1161/01.res.87.4.323

[56] McCarron RM, Chen Y, Tomori T, Strasser A, Mechoulam R, Shohami E, et al Endothelial-mediated regulation of cerebral microcirculation. J Physiol Pharmacol. 2006;57 Suppl 1: 133–44. 17244945

[57] Onaivi ES. Commentary: Functional Neuronal CB2 Cannabinoid Receptors in the CNS. Curr Neuropharmacol. 2011;9: 205–8. 10.2174/157015911795017416 21886591 PMC3137183

[58] Palazuelos J, Aguado T, Egia A, Mechoulam R, Guzmán M, Galve-Roperh I. Non-psychoactive CB2 cannabinoid agonists stimulate neural progenitor proliferation. FASEB J. 2006;20: 2405–7. 17015409 10.1096/fj.06-6164fje

[59] Stella N. Cannabinoid and cannabinoid-like receptors in microglia, astrocytes, and astrocytomas. Glia. 2010;58: 1017–1030. 10.1002/glia.20983 20468046 PMC2919281

[60] Amenta PS, Jallo JI, Tuma RF, Elliott MB. A cannabinoid type 2 receptor agonist attenuates blood-brain barrier damage and neurodegeneration in a murine model of traumatic brain injury. J Neurosci Res. 2012;90: 2293–305. 10.1002/jnr.23114 22903455

[61] Zhang M, Martin BR, Adler MW, Razdan RK, Ganea D, Tuma RF. Modulation of the balance between cannabinoid CB(1) and CB(2) receptor activation during cerebral ischemic/reperfusion injury. Neuroscience. 2008;152: 753–60. 10.1016/j.neuroscience.2008.01.022 18304750 PMC2577828

[62] García-Gutiérrez MS, Ortega-Álvaro A, Busquets-García A, Pérez-Ortiz JM, Caltana L, Ricatti MJ, et al Synaptic plasticity alterations associated with memory impairment induced by deletion of CB2 cannabinoid receptors. Neuropharmacology. 2013;73: 388–96. 10.1016/j.neuropharm.2013.05.034 23796670

[63] De Jesús ML, Hostalot C, Garibi JM, Sallés J, Meana JJ, Callado LF. Opposite changes in cannabinoid CB1 and CB2 receptor expression in human gliomas. Neurochem Int. 56: 829–33. 10.1016/j.neuint.2010.03.007 20307616

[64] Barzó P, Marmarou A, Fatouros P, Hayasaki K, Corwin F. Contribution of vasogenic and cellular edema to traumatic brain swelling measured by diffusion-weighted imaging. J Neurosurg. 1997;87: 900–7. 9384402 10.3171/jns.1997.87.6.0900

[65] Cernak I. Animal models of head trauma. NeuroRx. 2005;2: 410–22. 16389305 10.1602/neurorx.2.3.410 PMC1144485

[66] Ding JY, Kreipke CW, Speirs SL, Schafer P, Schafer S, Rafols JA. Hypoxia-inducible factor-1alpha signaling in aquaporin upregulation after traumatic brain injury. Neurosci Lett. 2009;453: 68–72. 10.1016/j.neulet.2009.01.077 19429018 PMC2703426

[67] Kent S, Bluthé RM, Kelley KW, Dantzer R. Sickness behavior as a new target for drug development. Trends Pharmacol Sci. 1992;13: 24–8. 1542935 10.1016/0165-6147(92)90012-u

[68] Larson SJ, Dunn AJ. Behavioral effects of cytokines. Brain Behav Immun. 2001;15: 371–87. 11782104 10.1006/brbi.2001.0643

[69] Potokar M, Stenovec M, Jorgačevski J, Holen T, Kreft M, Ottersen OP, et al Regulation of AQP4 surface expression via vesicle mobility in astrocytes. Glia. 2013;61: 917–28. 10.1002/glia.22485 23505074

[70] Vizuete ML, Venero JL, Vargas C, Ilundáin AA, Echevarría M, Machado A, et al Differential upregulation of aquaporin-4 mRNA expression in reactive astrocytes after brain injury: potential role in brain edema. Neurobiol Dis. 1999;6: 245–58. 10448052 10.1006/nbdi.1999.0246

[71] Lopez-Rodriguez AB, Acaz-Fonseca E, Giatti S, Caruso D, Viveros M-P, Melcangi RC, et al Correlation of brain levels of progesterone and dehydroepiandrosterone with neurological recovery after traumatic brain injury in female mice. Psychoneuroendocrinology. 2015;56: 1–11. 10.1016/j.psyneuen.2015.02.018 25770855

